# Using Crisis Theory in Dealing With Severe Mental Illness–A Step Toward Normalization?

**DOI:** 10.3389/fsoc.2022.805604

**Published:** 2022-06-09

**Authors:** Johanna Baumgardt, Stefan Weinmann

**Affiliations:** ^1^Department of Psychiatry, Psychotherapy and Psychosomatic Medicine With FRITZ am Urban & Soulspace, Vivantes Hospital Am Urban und Vivantes Hospital im Friedrichshain, Charité–Universitätsmedizin Berlin, Berlin, Germany; ^2^Department of Psychiatry and Psychotherapy, Center for Psychosocial Medicine, University Medical Center Hamburg-Eppendorf, Hamburg, Germany; ^3^University Psychiatric Hospital Basel, Basel, Switzerland

**Keywords:** psychiatrization, psychiatric diagnoses, *Recovery Model*, *Crisis Theory*, *Stress-Vulnerability Model*

## Abstract

The perception of mental distress varies with time and culture, e.g., concerning its origin as either social or medical. This may be one reason for the moderate reliability of descriptive psychiatric diagnoses. Additionally, the mechanisms of action of most psychiatric treatments and psychotherapeutic interventions are generally unknown. Thus, these treatments have to be labeled as mostly unspecific even if they help in coping with mental distress. The psychiatric concept of mental disorders therefore has inherent limitations of precision and comprises rather fuzzy boundaries. Against this background, many people question the current process of diagnosing and categorizing mental illnesses. However, many scholars reject new approaches discussed in this context. They rather hold on to traditional diagnostic categories which therefore still play a central role in mental health practice and research and. In order to better understand the adherence to traditional psychiatric concepts, we take a closer look at one of the most widely adopted traditional concepts – the *Stress-Vulnerability Model*. This model has originally been introduced to tackle some problems of biological psychiatry. However, it has been misapplied with the result of drawing attention preferentially to *biological* vulnerability instead of a wider array of vulnerability factors including social adversity. Thus, in its current use, the *Stress-Vulnerability Model* provides only a vague theory for understanding mental phenomena. Therefore, we discuss the advantages and allegedly limited applicability of *Crisis Theory* as an alternative heuristic model for understanding the nature and development of mental distress. We outline the problems of this theory especially in applying it to severe mental disorders. We finally argue that an understanding of *Crisis Theory* supported by a systemic approach can be applied to most types of severe psychological disturbances implying that such an understanding may prevent or manage some negative aspects of the psychiatrization of psychosocial problems.

## Introduction

Psychiatric terminology has become increasingly influential in the everyday lives of many people in the Western world; a growing body of evidence even suggests that we are witnessing a psychiatrization of society (Beeker et al., 2021). More and more, we view psychosocial phenomena in psychiatric terms and qualify them as objects for treatment (Frances, 2010). At the same time, there is no compelling evidence that the actual burden of suffering from psychological problems has increased substantially (Richter et al., 2019). Currently, in order to quantify psychiatric burden in population-based studies, one counts “treated morbidity” as well as problems that are not yet treated but being classified as psychiatric and therefore receive a commonly accepted psychiatric diagnosis (Cova Solar et al., 2020). This is a problematic approach since the definition of what counts as a mental health problem varies by time and culture, e.g., concerning the perception of psychological problems as either social or medical. Existing attributions range from mental crisis seen as a reaction to life events up to the occurrence of an – assumed – underlying mental health disorder that manifests under certain circumstances (Kleinman et al., 1978; Viswanath and Chaturvedi, 2012). Thus, it can be stated that orthodox psychiatric nosology as well as the process of current diagnostics are increasingly being challenged (Dean, 2012).

Amidst the different conceptualizations of what constitutes a psychiatric disorder and what not, the task of the clinical psychiatrist is to identify the correct – in the sense of best suitable – classification and to start treatment assuming that the applied interventions are disorder-specific. However, the latter assumption is built upon empirical efficacy studies with participants diagnosed according to the prevailing diagnostic guidelines at a particular time and against a certain cultural (Western) background (Fàbrega, 2001). Additionally, it has to be taken into account that the scientific basis for classification is weak. There are no usable biomarkers to detect or confirm psychiatric disorders or give us a hint for selecting or refining treatment interventions (de Leon, 2013; Margraf and Schneider, 2016). Additionally, there is also no tangible evidence of a concrete biological vulnerability factor predisposing to any classical psychiatric disorder. This also holds true for the so-called severe mental disorders such as schizophrenia (Fusar-Poli and Meyer-Lindenberg, 2016). For example, the etiology of schizophrenia was thought to be based on an excess of dopamine. This hypothesis falls short because it does not capture the complex influences and factors contributing to the mental experiences and behaviors associated with schizophrenia. Nevertheless, many scholars assume an underlying condition in the sense of a biological or neurodevelopmental disorder of the brain causing the symptoms. Such symptoms are thought to be based on an imbalance that cannot be cured but only corrected, compensated, or attenuated. Even if a severe psychological crises or severe mental disorder is thought to be triggered by life events (Beards et al., 2013), these events are seldom perceived as causal. Such an understanding of mental disorders not only entails prejudices as well as stigma but can also lead to unnecessary medicalization of psychosocial problems in general (Hyman, 2010; Pierre and Frances, 2016).

Considering the lack of biological tests, fuzzy boundaries, cultural influences on what people perceive as a mental health problem and further shortcomings, current psychiatric diagnoses can be seen as rather heuristic constructs instead of biological entities. This is exactly what the Diagnostic and Statistical Manual of Mental Disorders IV-text revision guidebook (DSM-IV-TR) states by saying that mental disorders defined are best conceived as “*valuable heuristic constructs”* rather than of “*well-defined entities that describe nature exactly as it is”* (Frances et al., 1995, p. 12). Thus, mental disorders can neither be objectified nor counted (Allsopp et al., 2019) – making e.g., epidemiologic numbers quite an imperfect proxy to *real* psychiatric morbidity (Thornicroft, 2007). While alternative models of psychiatric disorders like dimensional concepts or concepts of psychosocial crises of shorter or longer duration could be helpful at this point, many scholars are reluctant to use them. Except for common mental disorders or crises directly related to a specific stressful life event, they mostly reject such theories and their usefulness (Caplan, 1989). Even scholars who tried to adapt psychosocial theories such as the *Crisis Theory* to people with severe mental disorders emphasized the differences to “normal people” and asserted that the theory needs to be considerably modified to be helpful in this target group (Ball et al., 2005).

To better understand the rejection of psychosocial concepts, it appears helpful to take a closer look at the reasons for the strong adherence to traditional models of severe mental disorders. Hereby it is worthwhile to investigate not only psychiatric disorder concepts, but also heuristic models such as the *Stress-Vulnerability Concept* since it seems to bridge the gulf between what we do not know (etiology) and what we see (the so-called signs and symptoms).

## Conceptual Problems in Psychiatry: the Stress-Vulnerability Model

A seemingly comprehensive and useful model for explaining why some people become psychiatrically ill and others not, is the *Stress-vulnerability model* of Zubin and Spring (1977). This model has been welcomed widely on an international scale and used for decades. It was originally developed for a deeper understanding of schizophrenia. Over time, it was extended to other psychiatric diagnoses. Despite being criticized for not having included resilience as well as gene-environment interaction (Rutten et al., 2013), the *Stress-vulnerability model* appears to be consistent, acceptable, and adaptable for all professions dealing with people with psychological disorders over the past decades (Monroe and Simons, 1991).

### Background and Definition

The *Stress-vulnerability model* proposes that each human being is endowed with a genetic predisposition to stress – his or her individual mental vulnerability (Zubin and Spring, 1977). This vulnerability interacts with psychosocial stressors and results in a disruption to wellbeing and mental health. Vulnerability can be defined as “*the empirical probability that an individual will experience an episode of psychiatric disorder”* (Zubin and Spring, 1977, p. 123). The authors call this an “*enduring trait”* which affects the capacity to cope with external stressors or rather one's “*coping ability”* (Zubin and Spring, 1977, p. 123). Thus, individuals may experience a “*coping breakdown”* in the case of being exposed to substantial or even catastrophic stress. This breakdown does not necessarily lead to an episode of a psychiatric disorder. The originators of the model therefore argue that only individuals with a higher vulnerability experience coping breakdown episodes of time-limited or chronic illness (Zubin and Spring, 1977, p. 109). The diversity of possible variations in vulnerability is explained by the fact that an individual's degree of vulnerability is as well inherited and acquainted over the life span e.g., through trauma, disorders, perinatal complications, family experiences, adolescent peer interactions, and other life events. Consequences of such events are thereby compared to consequences of somatic events and therefore understood as something like a ‘neuro-psychiatric injury’ (Read et al., 2009).

### Original Conceptualization of Vulnerability

Externalizing causes of mental stress or making brain and function responsible for mental disorders was thought to relieve a person from possible feelings of guilt or shame – which in turn was expected to lower stigmatization of mental disorders. The authors of the *Stress-vulnerability model* also hoped that using the word “vulnerability” rather than “disorder” would help to regard individuals as suffering from a (hopefully) temporary episode rather than a chronic disorder (Zubin and Spring, 1977). Besides offering a plausible explanation of psychiatric episodes, the *Stress-vulnerability model* was also developed to guide interventions. Zubin and Spring (1977) offered two avenues for intervention: On one hand, they suggested that vulnerability can be “*reduced or inhibited from full-blown expression through psychopharmacological intervention”* (Zubin and Spring, 1977, p. 122). On the other hand, psychological interventions might be applied to “*restore coping ability or reduce the threatening nature of life events that produce the breakdown.”* (Zubin and Spring, 1977, p. 122). These suggestions as well as the later use of the model show that the authors already had the biological level in mind when they spoke of vulnerability. Such an alleged biological vulnerability in turn is comprised of e.g., putative genetic risks and changes in protein expression, structural and functional brain anomalies, neurochemical deficits, anomalies or particularities, impairments, problems of connectivity or neurons among others (Beauchaine et al., 2008; Sullivan et al., 2012).

### The Hegemony of Biological Vulnerability and Its Consequences

Combining biological, psychological and social aspects, the *Stress-vulnerability model* became the foundation of the so called ‘bio-psycho-social model of psychiatric disorders’. This model was welcomed widely by psychiatry (Engel, 1978; Engert et al., 2020). However, this model was of rather low additional informative value, because it did not move past the biomedical model in any meaningful way (Ghaemi, 2009). Additionally, it was frequently used as a pure slogan rather than actually integrated into a holistic understanding of mental disorders. It is e.g., argued that the ‘bio-psycho-social model of psychiatric disorders’ is mostly used as just a ‘bio-bio-bio’ or at least as a ‘bio-bio-psychological model of mental disorder’ (Read et al., 2009), granting a causal role to social factors but limiting them to being ‘causal chain links’ leading inexorably to biological processes. Such an understanding shows no significant difference to the biomedical model in any meaningful way and is therefore in line with the almost hegemonic biogenetic conceptual framework in understanding mental disorders (Malla et al., 2015).

Maintaining a biological understanding of psychological problems despite the above described brings about several problematic repercussions:

1. Without biomarkers, valid criteria and boundaries for psychiatric disorders, psychological suffering always bears a risk of being seen as a psychiatric problem. In the absence of objective standards of verification, it is almost impossible to establish what a specific disorder is and who is affected (Gupta, 2014, p. 86). Thus, it allowed psychiatrists e.g., to expand the *Stress-vulnerability model* to minor psychological problems with a certain amount of suffering, such as mild or moderate depression and consequently prescribe psychiatric treatments (Kinser and Lyon, 2014). In our view, this effect is aggravated by the widespread availability of biological treatments, which are not limited to severe disorders but are also prescribed in minor psychological crises. Even though such an approach might alleviate suffering for a certain individual, one has to keep in mind that a biased understanding and use of the *Stress-vulnerability model* together with the application of a biological understanding of psychological problems might contribute unintendedly as a ‘top-down factor to psychiatrization’ (Beeker et al., 2021). Top-down psychiatrization hereby refers to constructing and restructuring images of mental health by psychiatrists and researchers in order to put the problem into the context of a medical and medically treatable disorder – with a minor role of the social world in which the person affected and the problem are located.
2. Evidence shows that biological narratives are not linked to reduced blame (Loughman and Haslam, 2018). In fact, neurobiological or genetic explanations for psychiatric disorders seem to lead to an even greater desire for social distance, greater perceived dangerousness, and greater prognostic pessimism (Pescosolido et al., 2010). This in turn results in higher stigmatization of people with so-called severe mental illness – which was the exact opposite of what Zubin and Spring (1977) originally intended.
3. A primarily biological understanding of mental crisis ignores existing evidence of the central importance of the social context that might be associated with relational stress and increasing the vulnerability to psychiatric disorders e.g., in the case of psychosis (Longden and Read, 2016; Jongsma et al., 2021).
4. Despite a lack of evidence, the dominant biological narrative increased the use of psychiatric medication since they are claimed – among other effects – to decrease vulnerability. Additionally, there is increasing evidence about short and long-term side effects e.g., even of modern antidepressants and antipsychotics (Moncrieff, 2006; Kendall, 2011; Davies et al., 2019). Particularly antidepressants carry a high level of risk of withdrawal and rebound phenomena (Henssler et al., 2019; Lerner and Klein, 2019), which in turn is not in line with its effectiveness narrative. In addition, there is considerable evidence that antidepressants prescribed over a longer term worsen the outcome of depression (Fava, 2003).
5. A focus on biological vulnerability might unnecessarily lead to neglecting the psychosocial aspect concerning the care of people with severe psychological symptoms, e.g., regarding research and the application of interventions. There is evidence that such a focus e.g., leads to a smaller consideration of biographic and adverse life events in the which in turn is associated with oftentimes unnecessarily and sometimes very aggressive pharmacological interventions for too long and at a too high dosage (Paris, 2017). This might not only increase stigmatizing attitudes in professionals but also undermine self-healing powers in patients and might push them to adopt the biological model to themselves (Lebowitz and Appelbaum, 2019).
6. A biological understanding fails to provide early psychosocial interventions because it suggests a correction of biological vulnerability before psychosocial measures can be applied “in addition.” Thus, whenever a situation occurs in which a person's behavior or reported internal world resembles a classification of the DSM or ICD, we assume a biological vulnerability. Consequently, we tend to look for biological remedies to alleviate it. In addition, psychosocial interventions are oftentimes implemented with the aim to strengthen the individual's coping ability, resilience, or acceptance of the assumed “disorder” in order to improve the outcome (Ross, 2014). This simplified understanding of the *Stress-vulnerability model* is the opposite of what its authors aimed at: They proposed the *Stress-vulnerability model* with the aim to substitute a mainly medical understanding of continuing psychiatric disorders such as schizophrenia with a holistic view of temporary episodes in vulnerable individuals whose problem is in the majority of cases self-curing (Zubin and Spring, 1977, p. 121-122).

## An Alternative Model for Mental Distress: the Crisis Theory

As outlined above, in its current use, the *Stress-vulnerability model* gives preference to biological narratives and remedies and only provides a vague and rather reductionist theory to understand minor as well as major psychological and social phenomena and disturbances. Other models such as *Crisis Theory* (Caplan, 1964; Hobbs, 1984) may better convey the process and nature of mental crises and offer a more inclusive approach to dealing with them. Furthermore, in our view, *Crisis Theory* represents an important tool in preventive psychiatry since it provides a conceptual framework for an increasing number of community-based multidisciplinary psychiatric services.

### Background, Definition and Location in Psychiatry

Arising from non-medical disciplines, *Crisis Theory* was originally proposed by Caplan and Hobbs (Caplan, 1964; Hobbs, 1984). It accounts as an explicitly *descriptive* and coherent explanatory model in which the experience of a crisis as a psychological phenomenon is per definition subjective. Hereby, a psychological crisis is defined as a substantial and critical incident that elicits a response to trauma (Hobbs, 1984). Key features of such a psychological crisis are the following: (a) an individualized life experience based on subjective appraisal; (b) acute distress related to feeling overwhelmed and without or only little control over the situation; (c) changes in the day-to-day social functioning abilities and risk behaviors; (d) the importance of social support as potentially protective as well as helpful to cope with the crisis (Hobbs, 1984; Dulmus and Hiarski, 2003). In contrast to an understanding of a mental disorder as being chronic and driven essentially by biology, crises are seen as temporal and episodic phenomena (particularly outside the psychiatric context). These phenomena are thought to be an opportunity for change or a turning point in the life of an individual (Hobbs, 1984). As such, a crisis can offer room for inner development as well as post-traumatic growth (Slaikeu, 1990). This focus on life events and development in turn offers explanations and entering points for interventions without using psychiatric labels: Changes in appraisal of events such as reappraisal of existing beliefs and values, changes in living circumstances, mobilization of social, psychological or financial resources, transitions etc. Against the outlined understanding of a crisis, such interventions maximize the potential for psychic growth and maturation.

### “A Close Relative”: The *Recovery Model*

A concept that shares some key features with the *Crisis Theory* and that has gained significantly in importance among both users of psychiatric services and service providers is the *Recovery Model* (Shepherd et al., 2008; Amering and Schmolke, 2009). The *Recovery Model* views mental disorders from a perspective that is radically different from traditional psychiatric approaches even though it does not fully explain why people develop psychological problems. The *Recovery Model* rather emphasizes resilience and control over problems and life in situations where some kind of shared experience with others and autonomy have been lost. To our knowledge, there is as yet no single definition of the concept of recovery for people with mental health issues. It is rather understood as a process, an outlook, a conceptual framework with certain guiding principles (Slade et al., 2014). These guiding principles emphasize hope and a strong belief that it is possible for people with mental health problems to regain a meaningful life despite persistent symptoms (ibid). Thereby the *Recovery Model* argues against just treating or managing symptoms but focusing on building resilience in people with mental health problems and supporting those in emotional distress. Thus, for many people, the concept of recovery is mostly about staying in control of their life rather than the elusive state of return to a premorbid level of functioning or an asymptomatic phase of the person's life (Davidson, 2005; Ramon et al., 2007; Bonney and Stickley, 2008; Jacob, 2015). Consequently, the *Recovery Model* implies an understanding of a psychological crisis as being a temporary phenomenon that does not necessarily become a chronic one and that does not have a life-long reference to a specific deficit called vulnerability. In this light, “vulnerability” can be understood as being mainly influenced by factors not inherent in one s genes, such as life events, loneliness, residual symptoms, social disadvantage, lack of social support, lack of sleep, drug consumption as well as conflicts (Ball et al., 2005).

### Crisis Theory and Severe Mental Illness

While many users of mental health services prefer a holistic understanding of mental crises, *Crisis Theory* as well as related concepts like the above mentioned *Recovery Model* are seldom fully explored among caregivers and service providers. Additionally, many psychiatrists seem pessimistic about the potential for recovery in people with psychiatric diagnoses (Jacob et al., 2017). We will therefore outline some problems and misunderstandings raised in this context and try to dissolve them:

1. *Crisis Theory cannot be applied widely in psychiatry because there are two distinct groups of people: the severely mentally ill who are victims of their biology and those suffering ‘mere’ distress in response to life events*.

Some people assume that individuals with severe and persistent mental illness are prone to crises even in the absence of clear external precipitants. They generally perceive a crisis as not lasting months or years, and they assert that it must be traceable to a specific life event. Against this, they argue that people affected by severe mental illness do often experience psychiatric symptoms not as a response to visible interpersonal crises but as a result of a neurobiological deficit which triggers acute episodes. They argue that *Crisis Theory* does not take adequately into account fluctuations in symptomatology over time. The view is that external stressful life events play a minor role in contributing to the pathogenesis of a psychiatric episode and that relapses and acute episodes are based on neurotransmitter disturbances, substance abuse, or medication non-compliance. In addition, with so-called severe mental illness, crisis concepts are rejected because, contrary to the commonly accepted crisis model it is often others who seek help on behalf of the individual in crisis, which does not fit with the theory's assumptions (Ball et al., 2005). Thus, they claim a limited application of *Crisis Theory* in mental health care (Ball et al., 2005). However, this is not an argument against *Crisis Theory* by itself but can be understood as a challenge to adapt classical *Crisis Theory* or one of its shortcomings. Coping with a crisis, either a classical psychological crisis as well as a severe crisis, always depends on previous environmental conditioning besides genetic imprinting. In addition, if staff as well as people with the experience of symptoms of so-called severe mental illness have been socialized by the medical community to medicalize these crisis experiences (Mak and Cheung, 2010), the connection of a severe crisis with external stress factors gets lost.

1. *Judgements in mental health care rely on a rather individualistic approach to psychological breakdowns without capturing the circumstances*.

In many psychiatric institutions, the focus of problem assessment and the starting point for its treatment is almost exclusively on the person with symptoms. In general, this approach does not capture the interpersonal nature of a crisis (Seikkula and Arnkil, 2016). To solve this shortcoming, some advocates of *Crisis Theory* refer to a systems theory approach. In this context, they suggest that an individual's psychological crisis can represent a crisis in the wider system. The bottom line of this construct is the assumption that an individual crisis does not happen in isolation but rather within a social context. Such a systemic approach suggests that phenomena framed as signs and symptoms, such as emotional expressions, thought disorder, anxiety or deviant behavior, should not only be seen as the visible parts in the pathogenesis of an individual disorder process but rather as responses of an individual embedded in a complex surrounding. Furthermore, a systemic perspective views crises as escalating vicious cycles of attempts to resolve a situation in which a threat is perceived (Fraser, 1998). The consequence of such a conceptualization is that the social context has to be taken into consideration when looking out for explanations of the crisis as well as in the endeavor of organizing support and mobilizing help.

1. *One has to make a distinction between a psychological crisis and a psychiatric emergency as part of a psychiatric acute episode*.

In general, typical acute psychiatric symptoms affecting the individual's basic mental functions and coping capacity may carry a risk of self-harm or harm for others. These symptoms determine and guide an emergency situation as well as its treatment. A psychosocial crisis, in turn, is primarily seen as stress-related. Applying the above outlined systemic perspective on *Crisis Theory*, there is no boundary between a psychiatric emergency and a psychosocial crisis even though the expression of the reaction to the external stressors is different in a severe psychiatric crisis. Cognitive and emotional stress, e.g., might be related to previous external stressors. Negative symptoms in a psychotic crisis, e.g., might be associated with avoidance of traumatic memories related to (previous) psychosis and hospitalization (Harrison and Fowler, 2004). Other psychopathological phenomena such as cognitive disorders might also not be explained by crises alone but might be psychological reactions in people with specific traumatic experiences and psychological patterns which have been developed in childhood (Schäfer and Fisher, 2011).

### Benefits of a Systemic Approach to Crisis Theory

As outlined above, a systemic perspective of *Crisis Theory* enables us to detect psychosocial problems behind the symptoms even in people with severe psychiatric crises. With such an understanding, these symptoms are part of a spectrum of “normal” responses within a dimensional and systemic perspective. In our view, a systemic perspective of *Crisis Theory* enables us to better identify precipitants and triggers of severe mental crises in the social context of those affected than, e.g., in a hospital setting, where people oftentimes are de-contextualized. Furthermore, a systemic crisis can be more easily addressed by crisis resolution, assertive outreach and home treatment teams as the psychosocial context is more visible within these approaches (Johnson et al., 2008). Since such an approach stresses the transient nature of crises, crisis intervention could act without having to identify and “treat” interpersonal conflicts immediately. This would on one hand release psychiatric staff from always being responsible to find a quick solution and on the other hand release the respective person whose ability to specify or speak about the precipitating social factors might be limited in an acute crisis.

A further benefit of applying a systemic approach of *Crisis Theory* is the fact that it is less prone to medicalization – which opens the possibility of using different approaches, remedies, interventions, or help from various fields of psychiatry in order to cope with or solve a mental health crisis (von Peter and Schwarz, 2021). Finally, a systemic approach of *Crisis Theory* does not put biological vulnerability and the search for it in the center, but focuses on the resources of the individual and his or her social context. Thus, it is a more inclusive approach of dealing with psychological phenomena (Schwarz et al., 2020).

## Discussion and Outlook

In this article, we argued that current psychiatric nosology lacks reliability as well as validity and is still based on unproven biological theories of mental disorders. Although the *Stress-Vulnerability Concept* has originally been developed to counteract a reductionist medical model of psychiatric disorders, its current use places assumed neuro-psychiatric injuries and resulting impairments due to an assumed psychiatric “disorder” on the same level as somatic disorders. In this sense, it seems to have been misapplied: Vulnerability has primarily been framed as biological. However, a primarily biological disorder model using a simple and one-dimensional *Stress-Vulnerability Concept* may contribute to psychiatrization of people in psychosocial stress situations, e.g., since certain ICD or DSM diagnoses in particular imply pharmacologic interventions. To stop this trend and contribute to a more inclusive, less stigmatizing, holistic way of dealing with mental health challenges, different concepts are needed. We suggest a broader *Crisis Theory* with a systemic perspective in which the individual crisis represents a crisis in the wider system. Applying it consequently could contribute to de-medicalize psychosocial suffering and might lead to a different perception. It could also enhance self-perception of mental health problems since these would rather be seen as challenges within a social system. Focusing on such a view could help to avoid dynamics of self-fulfilling prophecies when we speak of a “psychiatric disorder” and of potential “chronic disorders” – which we would have to avoid by using “adequate” treatment.

To ensure that such a model becomes reality, it not only has to be applied in common mental disorders but also for organizing care and support for people experiencing severe psychiatric episodes. Thus, we should not accept or ally ourselves with the concept of biological “otherness,” even when people have experienced relief with the help of medication or coercive measures. Such a narrow medical focus of disorder and treatment may result in alienating people from themselves including reducing their trust in themselves and their self-healing powers. Applying pharmaceutical intervention too fast and in too high dosages e.g., might lower the strength and energy of people affected to overcome their crisis– in a sense that the medication may alleviate their symptoms but leaves them with little creative energy to overcome the episode. Such a focus might also bias people in attributing emotional crises to the “disorder” which then might become a part of their personal identity.

Against the outlined in this article, we argue that *Crisis Theory* with a systemic perspective can be judged as very useful to overcome shortcomings of current psychiatric concepts, to empower people affected especially with regard to stating that you can recover even from so-called severe mental illness, to enhance the understanding that a psychological crisis may change your life without dominating it, to fight pessimism concerning recovery, to reduce stigmatization, and to strengthen the role of psychosocial interventions. Thereby, its application could help to prevent or manage some negative aspects of the psychiatrization of psychosocial problems.

## Author Contributions

SW drafted the first version of the manuscript. Both authors revised the manuscript several times, read, and approved the final version of the manuscript.

## Conflict of Interest

The authors declare that the research was conducted in the absence of any commercial or financial relationships that could be construed as a potential conflict of interest.

## Publisher's Note

All claims expressed in this article are solely those of the authors and do not necessarily represent those of their affiliated organizations, or those of the publisher, the editors and the reviewers. Any product that may be evaluated in this article, or claim that may be made by its manufacturer, is not guaranteed or endorsed by the publisher.
